# Label-Free Quantitative Proteomic Analysis of Chitosan Oligosaccharide-Treated Rice Infected with Southern Rice Black-Streaked Dwarf Virus

**DOI:** 10.3390/v9050115

**Published:** 2017-05-18

**Authors:** Anming Yang, Lu Yu, Zhuo Chen, Shanxue Zhang, Jing Shi, Xiaozhen Zhao, Yuanyou Yang, Deyu Hu, Baoan Song

**Affiliations:** 1 State Key Laboratory Breeding Base of Green Pesticide and Agricultural Bioengineering/Key Laboratory of Green Pesticide and Agricultural Bioengineering, Ministry of Education, Guizhou University, Guiyang 550025, China; yam2415@163.com (A.Y.); yuji570@163.com (L.Y.); gychenzhuo@aliyun.com (Z.C.); 18985418398@163.com (J.S.); xuezai@163.com (X.Z.); 18354235351@163.com (Y.Y.); 2 Hainan ZhengyeZhongnong High Techchnolngy Co., Ltd/National Joint Engineering Laboratory of marine biological pesticide discovery, Haikou 570206, China; 18302647125@163.com

**Keywords:** chitosan oligosaccharide, Southern rice black-streaked dwarf virus, label-free proteomics, mechanism, defensive reaction, catalase

## Abstract

Southern rice black-streaked dwarf virus (SRBSDV) has spread from thesouth of China to the north of Vietnam in the past few years and severelyinfluenced rice production. Its long incubation period and early symptoms are not evident; thus, controlling it is difficult. Chitosan oligosaccharide (COS) is a green plant immunomodulator. Early studies showed that preventing and controlling SRBSDV have a certain effect and reduce disease infection rate, but its underlying controlling and preventing mechanism is unclear. In this study, label-free proteomics was used to analyze differentially expressed proteins in rice after COS treatment. The results showed that COS can up-regulate the plant defense-related proteins and down-regulate the protein expression levels of SRBSDV. Meanwhile, quantitative real-time PCR test results showed that COS can improve defense gene expression in rice. Moreover, COS can enhance the defense enzymatic activities of peroxidase, superoxide dismutase and catalase through mitogen-activated protein kinase signaling cascade pathway, and enhance the rice disease resistance.

## 1. Introduction

Southern rice black dwarf disease is one of the most serious plant diseases worldwide, mainly in East Asia and Southeast Asia, including China, Japan and Vietnam, and results in severe losses in grain production in these areas [1,2]. Controlling this disease is difficult, because its early symptoms arenot obvious and the viral latency islong. At present, the main method of control is using associated chemical pesticides combined with insecticides [3]. However, excessive use of chemical pesticides worsens environmental degradation to a certain extent and presents environmental hazards.

Chitosan oligosaccharide (COS) is an environmentally friendly biological regulator, which can be obtained by hydrolyzing naturally occurring chitosan [4]. It is a type of clean and good biological pesticide source that does not pollute the environment. In 1980, COS was first reported to have the ability to induce plant immunity [5]. Since then, many studies have shown that COS can induce the immune resistance of plants [6,7,8]. Furthermore, COS has been considered as a potent elicitor of plant immunity and thus is used in many plants, such as tobacco [9], wheat [10], camellia (*Camellia pitardii*) [11], oilseed rape resistance [12], tomato [13], and Arabidopsis [14,15]. At present, studies on the regulatory mechanism of COS mainly focus on receptor protein and signal transduction pathway discovery. In particular, receptor proteins are mostly found in cabbage leaves and bamboo leaves and these receptor proteins were found to be diverse [16,17]. The signal transduction process of anthraquinone were detected by stimulating COS in *Rubia tinctorum* L. cells. The results showed that the COS treatment groups can significantly improve the content of anthraquinone in *Rubia tinctorum* L. cells. The regulation process may be achieved by activating the MAPK signaling pathway and calcium messenger signaling pathway [18,19,20,21]. Meanwhile, numerous studies showed that COS can improve rice disease resistance [22,23] and enhance the disease resistance of southern rice black-streaked dwarf virus (SRBSDV)-infected rice [24]. However, the mechanism of COS-induced immunity in rice, especially the signaling processes involved, remains unclear.

In this study, label-free proteomics technique was performed to investigate the differences of proteins level in COS-treated rice infected with SRBSDV. The results showed that the COS preventing controlling mechanism may be determined through the improved activity of the relevant defensive enzyme, especially catalase, which is a key protein that activates the disease resistance of rice. This result provided the basis for subsequent studies on mechanisms that COS enhanced resistance against SRBSDV in rice.

## 2. Materials and Methods

### 2.1. Chitosan Oligosaccharide to Promote Rice Rooting and Germination

Japonica Rice Cultivar seeds Nipponbare (*Oryza sativa Japonica*) were randomly divided into four groups, namely, control check (CK), Treatment 1 (T1), Treatment 2 (T2), and Treatment 3 (T3). Water (150 mL) was added to the CK group, and 150 mL of COS solution was added to each of the other groups. The final concentrations of COS were 50, 100, and 200 µg/mL. The rice seeds were soaked for 12 h, and then the solution was removed until its germination. The germination rate, root length, and bud length of each group were then counted. Each experimental group was repeated three times.

### 2.2. Plant Material and Samples of Proteomics

Japonica Rice Cultivar seeds Nipponbare (*Oryza sativa Japonica*) were rinsed five times in clean water and germinated in plastic trays lined with wet paper towels for 72 h in the dark (25 °C). The seedlings were grown in illumination incubator under controlled conditions (28 °C/30 °C during the day cycle, relative humidity of 50%). When the third leaf was fully expanded after two weeks of culture, the seedlings were transplanted into another illumination incubator to inoculate the virus through SRBSDV-carrying white-backed plant hopper. After one week, the rice was transplanted into the experimental fields. We waitedfor the treatment groups and control groups to grow to the tiller period. COS (0.05 mg/mL) was sprayed on the rice plants under the treatment groups, andclean water was sprayed on the control groups. The samples were collected at 1, 3, 5, and 7 days after treatment and maintained at −80 °C for further use. The treatment and control groups were imposed in randomized design with three replicates.

### 2.3. Extraction of Total Proteins and Polyacrylamide Gel Electrophoresis

Total rice proteins were extracted and separated by polyacrylamide gel electrophoresis (PAGE) according to a modified method, which was reported previously [25,26]. First, approximately 1 g of rice sample was homogenized using mortar and pestle in liquid nitrogen until a fine powder is obtained. The total soluble proteins were then extracted using 5 mL of ice-cold protein extraction buffer containing 0.5 M Tris-HCl (pH 7.5), 0.7 M sucrose, 0.1 M KCl, 50 mM ethylenediaminetetraacetic acid (EDTA), and 40 mM dithiothreitol (DTT) at room temperature for 15 min. An equal volume of Tris-phenol was then added to an extraction tube after 30 min of shaking, and homogenates were centrifuged at 8000 g and 4 °C for 5 min. The supernatant was collected and added with five times volume of 0.1 M ammonium acetate in methanol. The liquid sample was maintained at −20 °C overnight and then centrifuged at 8000 g for 10 min at 4 °C. Finally, the resulting pellets were washed with ice-cold acetone containing 1% (*w*/*v*) DTT, and the washing step was repeated three times. The final pellets were dried in a vacuum drier for 2 h and then dissolved in 100 µL of the rehydration solution containing 8 M (*w*/*v*) urea, 0.1 M (*w*/*v*) Tris, and 10 mM DTT, and total protein concentration was determined through the Bradford method [27]. Each experiment was repeated three times. Gel electrophoresis was performed at 120 V, and the gel was visualized with colloidal Coomassie blue. The gel is divided into three segments according to the protein maker.

### 2.4. In-Gel Digestion

Proteins digestion with trypsin were performed as follows: the gels were briefly washed with buffer containing 50% (*w*/*v*) ACN and 50% (*w*/*v*) 100 Mm NH_4_HCO_3_. This step was repeated until the colors of the gels faded. The gels were then incubated at a solution of 10 mM of DTT (Sigma Aldrich (Shanghai) Trading Co., Ltd, Shanghai, China)/50 mM of NH_4_HCO_3_ (pH 8.0) for 1 h at 56 °C. Finally, the gel pieces were again incubated with a solution containing 55 mM iodoacetamide (Sigma-Aldrich)/50 mM of NH_4_HCO_3_ (pH 8.0) at dark condition for alkylation at room temperature for 30 min before removing the solution. The gel pieces were alternately washed with 10 mM NH_4_HCO_3_ and 100% ACN two times. The gel pieces were minced and allowed to dry before they were rehydrated in overdose trypsin (sequencing grade, Roche Diagnostics) in 10 mM NH_4_HCO_3_ at 37 °C overnight. The trypsin solution was added to equal volumes of 60% ACN/5% formic acid (FA) solution. The trypsin peptides were extracted from the gel grains with 0.1% FA in H_2_O (HPLC grade) three times and then dried using Speed Vac (SIM International Group Co, Ltd., Newark, NJ, USA). The precipitate was dissolved in 50 µL of H_2_O (HPLC grade) with 0.1% FA for liquid chromatograph-mass spectrometer/mass spectrometer(LC-MS/MS) analysis.

### 2.5. LC-MS/MS, Database Searching, and Bioinformatics Analysis

The peptide samples, which come from hydrolyzed gels, were analyzed using a Nano LC-1DTM plus system (Eksigent, Dublin, CA, USA) combined with TripleTOF 5600 MS (AB SCIEX, Foster City, CA, USA). Peptide samples (8 µL) were performed using a full loop injection. First, the peptide samples were desalted on a ChromXP Trap column (Nano LC TRAP Column, 3 µm C_18_-CL, 120 A, 350 µm × 0.5 mm, Foster City, CA, USA) and then eluted into a second analytical column-NanoLC C18 reversed-phase column (3C_18_-CL, 75 µm × 15 cm, Foster City, CA, USA). The mobile phases were composed of A mobile phase (5% ACN, 0.1% FA) and B mobile phase (95% ACN, 0.1% FA) over 120 min at a flow rate of 300 nL/min. TripleTOF 5600 MS was operated in the data-dependent mode to automatically switch between TOF–MS and Product Ion acquisition using Analyst (R) Software (TF1.6) (AB SCIEX, Foster City, CA, USA).β-Galactosidase digest was used to calibrate every two samples by 10 min of elution and 30 min of identification.

Raw data were processed using MaxQuant version 1.5.2.8 (Max Planck Institute, Munich, Germany), searched against a database, which contains data on rice, SRBSDV proteome, and 140,784 proteins and was downloaded from UniProt database (http://www.uniprot.org/); the search was performed on Andromeda search enginethat can be freely available at (www.maxquant.org) [28,29]. The search parameters were set as follows: the search of precursor mass tolerance of 20 ppm was used for mass recalibration. The search also included the variable modification of methionine oxidation and N-terminal acetylation and fixed modification of carbamidomethyl cysteine. In the main Andromeda search, precursor mass and fragment mass had initial mass tolerances of 6 and 20 ppm, respectively. For the identification of peptides and proteins, false discovery rate (FDR) was set to 0.01, and only significant peptides were accepted for the identification of the protein sample. For the comparison of the difference between the expression levels of the two groups, namely, control groups and treatment groups, label-free quantification with a minimum of two ratio counts was used to determine normalized protein intensity. The ratio and statistical analysis of the data met the requirements, that is, two instancesof repeated data appeared more than three times in the same group. Protein identifications were filtered to eliminate the identifications from the common contaminants and reverse database. Two-sample unpaired *t*-test was performed to identify the differentially accumulated proteins of the control and treatment groups. The intensity based absolute quantification (iBAQ) value, which is a protein quantitative method, was used for the *t*-test, and the proteins with a *p* of <0.05 in the Analysis of Variance (ANOVA) were considered differentially expressed.

The selected protein was used to cluster the samples in different groups to test the rationality and accuracy of differentially expressed proteins. Gene Ontology (GO) is a gene function in a standardized classification system and provides a dynamic update of the standardized vocabulary. Its aim is to describe the properties of genes and gene products on the basis of three aspects, namely, biological process (BP), cellular components (CC), and molecular functions (MF) [30,31]. All the rice proteins were used as backgrounds through the Fisher’s exact test to identify the differentially expressed proteins in the significant enrichment of GO functional item analysis and awareness of differentially expressed proteins and functional categories and to correlate them [32]. We analyzed and identified the most significant metabolic and signal transduction pathways of the differentially expressed proteins through theKyoto Encyclopedia of Genes and Genomes(KEGG) pathway annotation of the selected differentially expressed proteins [33]. The Search Tool for the Retrieval of Interacting Genes (STRING) database is an online tool designed to evaluate Protein-Protein Interactions (PPI) information (http://www.string-db.org/) [34]. We mapped some selected essential proteins to STRING and only the experimentally validated interactions with a combined score of more than 0.4 were considered significant.

### 2.6. Defense Enzyme Activity Assay

For the determination of Peroxidase (POD), Superoxide Dismutase (SOD), and Catalase (CAT) enzyme activities, 1 g of each rice sample was homogenized using a mortar and pestle and liquid nitrogen until fine powder was obtained. The obtained powder was ground in 1 mL of extraction buffer and centrifuged at 8000 rpm at 4 °C. Protein activity was determined using the Bradford protein assay kit (Sangon Biotech, Sanghai, China). Enzyme activity was calculated using an enzyme assay reagent kit according to the instruction manual (Suzhou Comin Bioengineering Institute, Jiangshu, China). Absorption wavelength was detected by Synergy H1 microplate reader (Bio-Rad, Hercules, CA, USA). Each experiment was repeated three times.

### 2.7. Gene Expression Analysis by Reverse Transcription-qPCR

Total RNA was extracted using a Trizol reagent kit (TakaRa, Dalian, China). RNA was reverse-transcribed using a cDNA kit (TakaRa) according to the manufacturer’s instructions. The experiments were performed in 10 µL of reaction volume and SYBR Premix Ex TaqII (TakaRa) and an iCycleriQ multi-color real-time PCR Detection System (Bio-Rad, California, CA, USA) wasused. Primer sequence information is listed in Table 1. Gene expression was normalized using β-actin as internal control. The relative copy numbers of the genes were calculated through the 2^−ΔΔCt^ method [35].

## 3. Results

### 3.1. COS to Promote Growth Results

Different COS concentrations promoted rice growth and the results indicated that lower concentration can promote rice rooting, but excessive concentration is not conducive to rice rooting. In the process of promoting budding growth, 50 and 100 µg/mL of COS facilitated germination. When rooting and germination is considered, lower COS concentrations were found to be conducive for plant growth experimentsin Table 2.

### 3.2. Label-Free ProteomicsComparative Analysis of Treatment Group and Control Group

#### 3.2.1. Analysis of Proteomics

**Figure 1:**
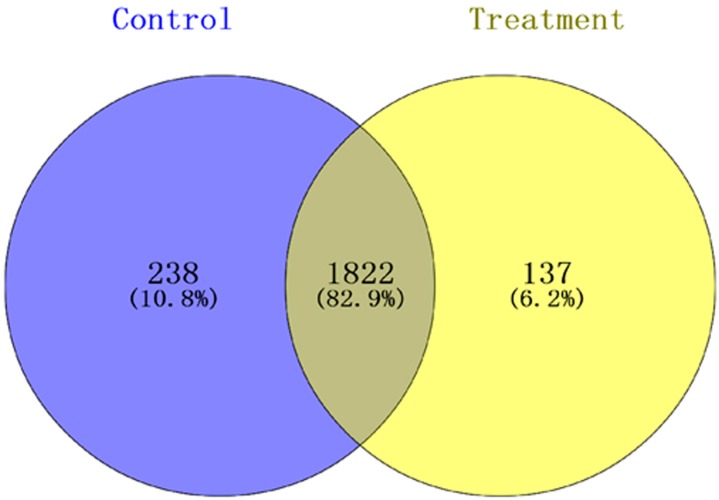
Venn diagram for proteins identified in the treatment and control groups. In the total protein, 2197 were identified in the two groups, of which 137 and 238 proteins are shown in theyellow part and blue part, respectively, and these were specifically expressed in the treatment and control groups, respectively.

**Figure 2:**
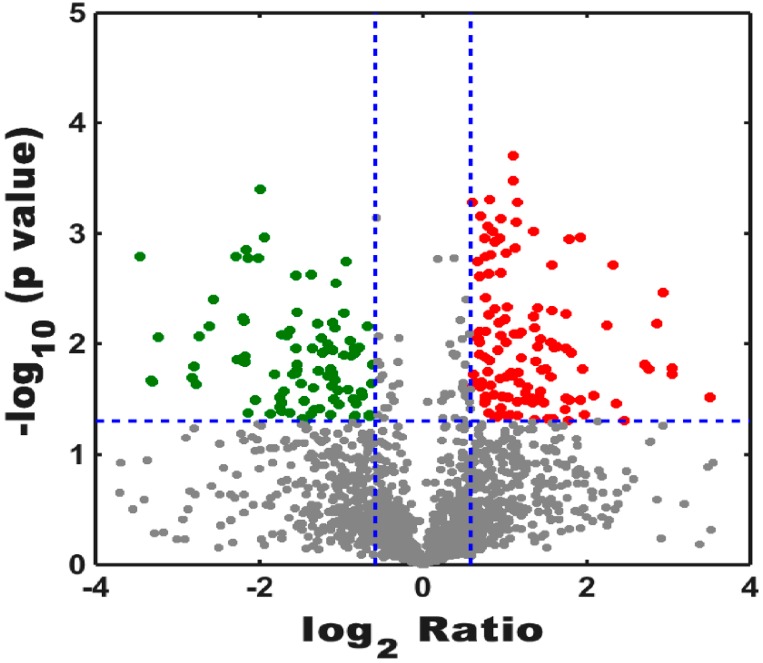
Numbers of identified proteins showed up-regulation and down-regulation among the control and treatment groups. Red spots represented up-regulation of proteins, and blue dots were down-regulated proteins. Log_2_ Ratio indicated that the ratio between the treatment groups and the control groups took the natural logarithm. The longitudinal coordinates indicate the magnitude of differences in protein level.

Peptide results were searched using MaxQuant version 1.5.2.8 (Max Planck Institute, Munich, Germany), and the result showed that 2197 proteins were identified and quantified when the selected filter settings in the supporting information (Table S1) were used. A total of 1959 and 2060 proteins were found in the treatment groups and control groups, respectively. In the total proteins, 2197 proteins were identified in the two groups, and 137 and 238 proteins were specifically expressed in the treatment groups and the control groups, respectively (Figure 1). To understand the expression levels of these differential proteins, we plotted a volcanic map (Figure 2), called the volcano plot. The total numbers of the identified different proteins, which included 124 up-regulated proteins (red dots) and 96 down-regulated proteins (blue dots), (fold change > 1.5, *p* < 0.05) in the control and treatment groups was 220 (for more information see Supplementary Materials Table S2).

#### 3.2.2. Bioinformatics Analysis of Control Groups and Treatment Groups

**Figure 3:**
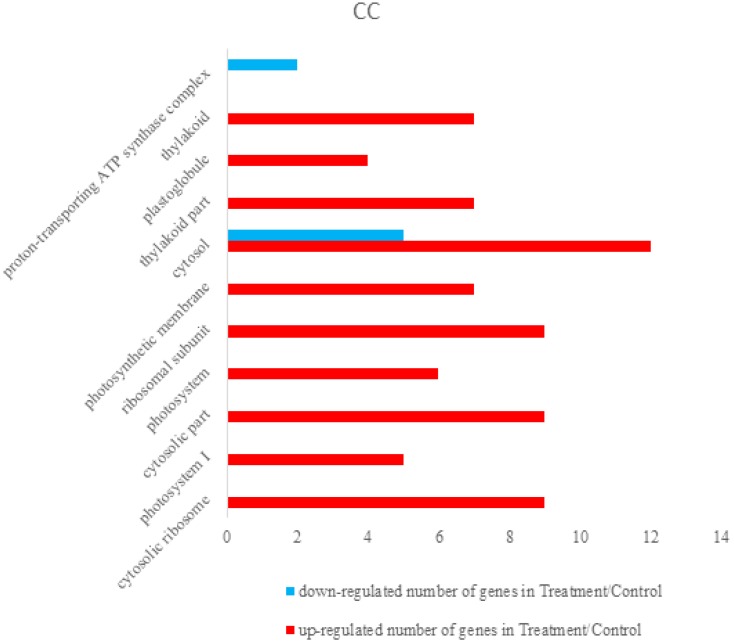
Some differently expressed proteins were grouped according to cellular components (CC), up-regulated proteins involved in cytosolic ribosome, photosystem I, cytosolic part, photosystem, ribosomal subunit, photosynthetic membrane, cytosol, thylakoid part, plastoglobule, and thylakoid. Down-regulated proteins only included cytosol and proton-transporting ATP synthase complex. Cytosol was mapped in the up-regulated and down-regulated proteins.

**Figure 4:**
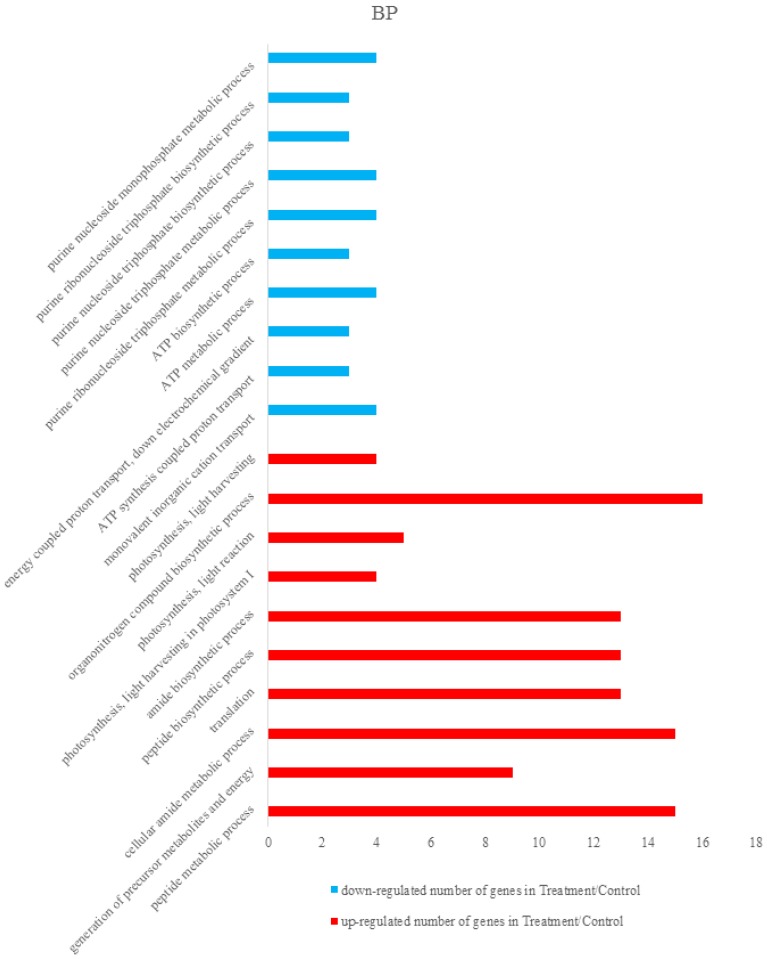
Some differently expressed proteins were grouped according to Biological process (BP). Some up-regulated proteins were involved in the peptide metabolic process, generation of precursor metabolites and energy, cellular amide metabolic process, translation, peptide biosynthetic process, amide biosynthetic process, photosynthesis, light harvesting in photosystem I, photosynthesis, light reaction, organonitrogen compound biosynthetic process, photosynthesis, light harvesting and partial down-regulated proteins mainly involved in monovalent inorganic cation transport, ATP synthesis coupled proton transport, energy coupled proton transport, down electrochemical gradient, ATP metabolic process, ATP biosynthetic process, purine ribonucleoside triphosphate metabolic process, purine nucleoside triphosphate metabolic process, purine nucleoside triphosphate biosynthetic process, purine ribonucleoside triphosphate biosynthetic process, and purine nucleoside monophosphate metabolic process.

**Figure 5:**
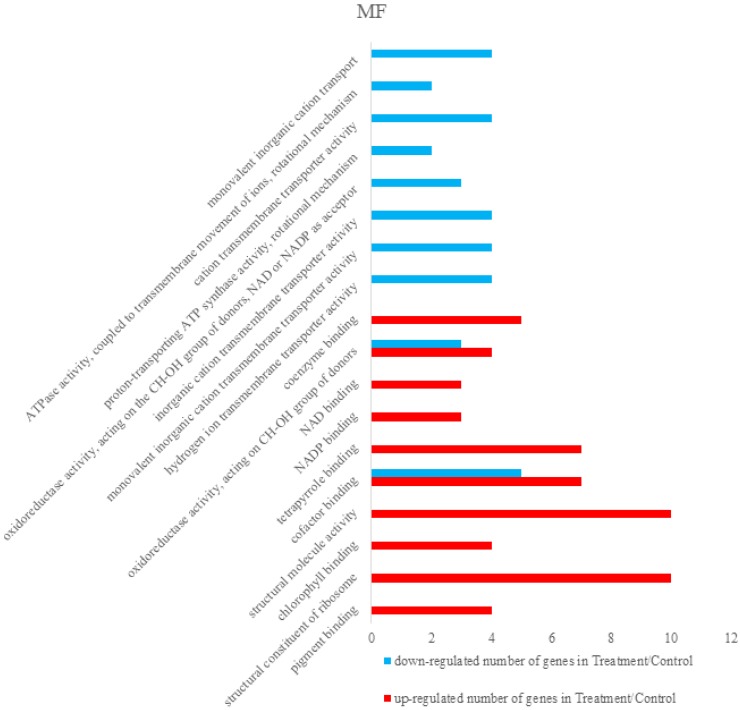
Proteins of molecular functions (MF) were mapped to pigment binding, structural constituent of ribosome, chlorophyll binding, structural molecule activity, cofactor binding, tetrapyrrole binding, nicotinamide adenine dinucleotide phosphate (NADP) binding, nicotinamide adenine dinucleotide (NAD) binding, oxidoreductase activity, acting on CH-OH group of donors, coenzyme binding, and down-regulate proteins were mapped to cofactor binding, tetrapyrrole binding, NADP binding, NAD binding, oxidoreductase activity, acting on CH-OH group of donors, coenzyme binding, hydrogen ion transmembrane transporter activity, monovalent inorganic cation transmembrane transporter activity, inorganic cation transmembrane transporter activity. Oxidoreductase activity, acting on the CH-OH group of donors, NAD or NADP as acceptor, proton-transporting ATP synthase activity, rotational mechanism and cation transmembrane transporter activity and ATPase activity, coupled to transmembrane movement of ions, rotational mechanism.

Differentially expressed proteins were annotated using The Database for Annotation, Visualization and Integrated Discovery 6.8 (DAVID 6.8) [36,37]. Total differentially expressed proteins were annotated with all proteins of rice (*Oryza sativa Japonica*) as background. The identified differentially expressed proteins were analyzed on the basis of GO categories in CC, BP, and MF through Fisher exact test and false discovery rate (FDR) correction method [38,39]. All the results of the differential proteins annotation are listed in the Supplementary Materials (Table S3). Some GO comments are listed according to the *p* value. The smallest ten *p* values are shown in the column chart. Figure 3 shows that differently expressed proteins were grouped according to: CC, up-regulated proteins mainly involved in cytosolic ribosome, photosystem I, cytosolic part, photosystem, ribosomal subunit, photosynthetic membrane, cytosol, thylakoid part, plastoglobule, and thylakoid. Down-regulated proteins only included cytosol and proton-transporting ATP synthase complex. Figure 4 demonstrated that up-regulated proteins are involved in peptide metabolic process, generation of precursor metabolites and energy, cellular amide metabolic process, translation, peptide biosynthetic process, amide biosynthetic process, photosynthesis, light harvesting in photosystem I, photosynthesis, light reaction, organonitrogen compound biosynthetic process, photosynthesis, light harvesting, down-regulated proteins mainly involved in monovalent inorganic cation transport, ATP synthesis coupled with proton transport, energy coupled proton transport, down electrochemical gradient, ATP metabolic process, ATP biosynthetic process, purine ribonucleoside triphosphate metabolic process, purine nucleoside triphosphate metabolic process, purine nucleoside triphosphate biosynthetic process, purine ribonucleoside triphosphate biosynthetic process, and purine nucleoside monophosphate metabolic process. They were grouped according to BP. The pigment binding, structural constituent of ribosome, chlorophyll binding, structural molecule activity, cofactor binding, tetrapyrrole binding, nicotinamide adenine dinucleotide phosphate (NADP) binding, nicotinamide adenine dinucleotide (NAD) binding, oxidoreductase activity, acting on CH-OH group of donors, and coenzyme binding were mapped to up-regulated proteins of MF, and the following were enriched to the down-regulated proteins of MF, as shown in Figure 5: cofactor binding, tetrapyrrole binding, NADP binding, NAD binding, oxidoreductase activity, acting on CH-OH group of donors, coenzyme binding, hydrogen ion transmembrane transporter activity, monovalent inorganic cation transmembrane transporter activity, inorganic cation transmembrane transporter activity, oxidoreductase activity, acting on the CH-OH group of donors, NAD or NADP as acceptor, proton-transporting ATP synthase activity, rotational mechanism and cation transmembrane transporter activity and ATPase activity, coupled to transmembrane movement of ions, and rotational mechanism.

### 3.3. Determination of Defense Enzyme Activity

**Figure 6:**
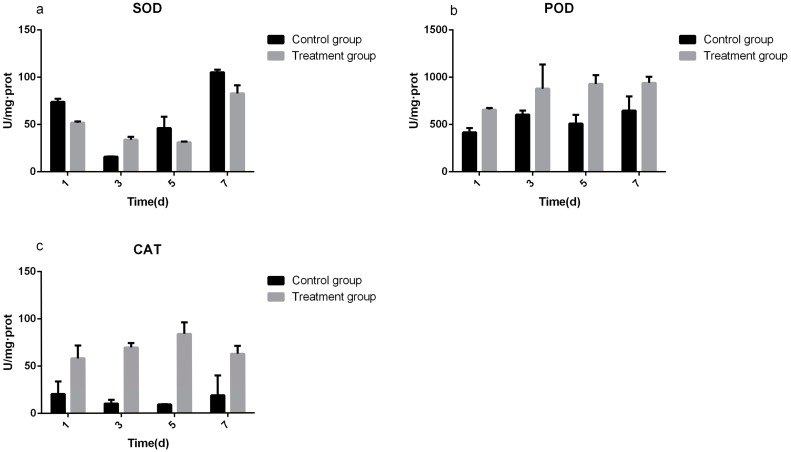
(**a**) Superoxide dismutase activities of COS treatment groups were 51.99, 33.93, 31.13, and 82.89 U/mg·protein (in 1, 3, 5, and 7 days, respectively). The control groups were 73.90, 15.90, 46.19, and 105.32 U/mg·protein (in 1, 3, 5, and 7 days, respectively). Only the third day is higher than the control groups. (**b**) The activities of Peroxidase in the treatment groups and control groups. The treatment groups were 658.37, 878.81, 929.02, and 940.74 U/mg·protein (in 1, 3, 5, and 7 days, respectively). The activities of control groups were 416.44, 604.58, 509.69, and 646.51 U/mg·protein (in 1, 3, 5 and 7 days, respectively). The treatment groups were 1.58, 1.45, 1.82, and 1.46 times higher than that of the control group. The activities of Catalaseare shown in (**c**), the treatment groups activities were 58.24, 69.61, 83.84, and 62.55 U/mg·protein, (in 1, 3, 5, and 7 days, respectively). They are higher than the control groups activities that were 20.50, 10.36, 9.19, and 18.71 U/mg·protein respectively. The fifth day activity had the biggest difference; the treatment group is 9.12 times of the control.

SOD is a kind of natural scavenger that can remove oxygen free radicals in organisms and it is an O^2−^ free radical quenchant. In the present study, COSinduced SOD expression in the rice samples, as indicated by the results shown in Figure 6a. The SOD activities of the COS-treated groups were 0.70, 2.13, 0.67, and 0.79 times of that of the control groups after 1, 3, 5, and 7 days, respectively. The POD activities in the treatment and control groups were detected at different time points (1, 3, 5, and 7 days). The results are shown in Figure 6b. The POD activities of the COS-treated groups were 658.37, 878.1, 929.02, and 940.74 U/mg·protein after 1, 3, 5, and 7 days, respectively. Control groups were 416.44, 604.58, 509.69, and 646.51 U/mg·protein at same time points. The treatment groups were 1.58, 1.45, 1.82, and 1.46 times that of the control groups after 1, 3, 5, and 7 days, respectively. The results indicated that CAT activity in the treatment groups increased first and then decreased, but remained higher than that of the control groups (Figure 6c). The treatment groups were 2.84, 6.72, 9.12, and 3.34 times that of the control groups after 1, 3, 5, and 7 days, respectively.

### 3.4. RNA Expression Levels of Chitinase, Catalase, Glutathione POD, Copper/Zine SOD, and PR-1

**Figure 7:**
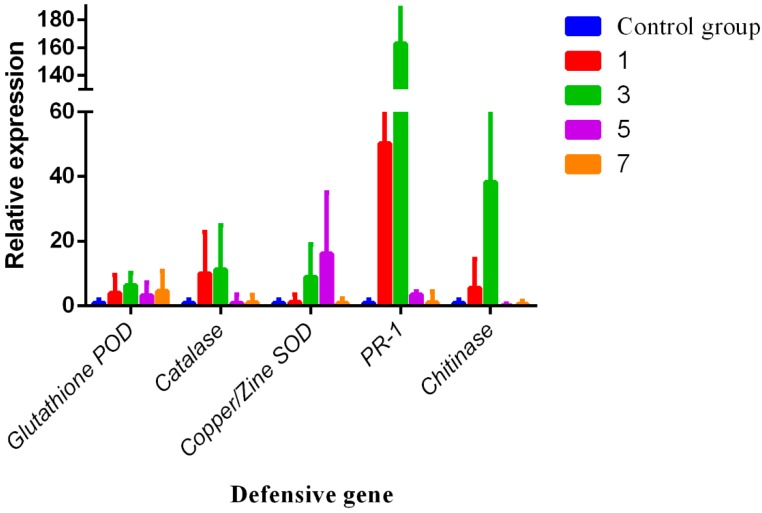
Gene expression of the defense genes were performed by RT-qPCR. The relative expression of defense genes, including *Chitinase*, *Catalase*, *Glutathione POD*, *SOD*, and *PR-1*, in different stages (1, 3, 5, and 7 days), were highly up-regulated by COS treatment.

Defense gene relative expression of *Chitinase*, *Catalase*, *Glutathione POD*, *Copper*/*Zine SOD*, and pathogenesis related protein 1 (*PR-1*) were investigated by reverse transcription (RT-qPCR). Results are shown in Figure 7. Results demonstrated that the relative expression levels of *Chitinase*, *Catalase*, *Glutathione POD*, *Copper*/*Zine SOD*, and *PR-1* of COS treatment groups were significantly higher than that of the control groups. The relative expression levels of *Glutathione POD* gene were 4.70, 5.40, 4.65, and 4.65 in 1, 3, 5, and 7 days, respectively, and the third day significant maximum reached 5.4. Meanwhile, the relative expression of *Catalase* also reached the maximum on the third day, after the COS treatment increased by approximately 12.13 times. Moreover, the relative expression of *Copper*/*Zine SOD*, *catalase*, and *PR-1* also were highly up-regulated by COS treatment in varying degrees and *PR-1* even reached approximately 172.85 times on the third day, but *Copper*/*Zine SOD* decreased after the third day.

## 4. Discussion

Plants have resistance to external stress when they are stimulated by external stimuli [40,41]. In this study, the change at the protein level was investigated in the rice of COS-treated groups and control groups by label-free quantitative proteomic technique. We annotated all different proteins in DAVID 6.8 (Supplementary Materials Table S3). Table 3 lists the primary differential proteins annotation results. After COS processing, we identified some defense-related proteins, simultaneously the expression of SRBSDV P9-1 and P5 protein were down-regulated [42,43]. Previous studies have shown that the interaction between P9-1 and P5 were essential proteins that formed viroplasms [44,45]. Our results indicated that COS may decrease the infection ability of SRBSDV.

**Figure 8:**
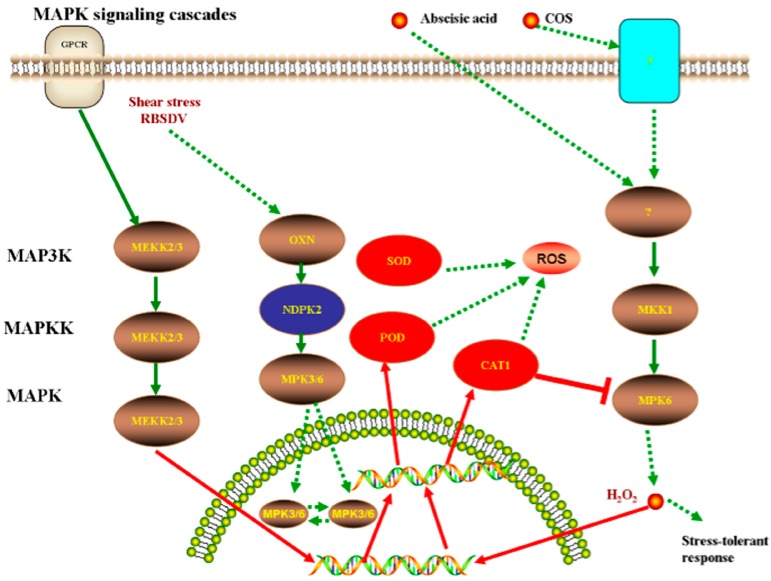
Catalase andnucleoside diphosphate kinase (NDPK2) played important regulative roles in mitogen-activated protein kinase (MAPK) signaling cascades pathway. The catalase can inhibit the production of H_2_O_2_, help cells to remove the harmful effects of reactive oxygen species, and result stress-tolerant response. NDPK2 that was only expressed in control groups, and due to the phosphorylation of MPK3/6 was not observed in treatment groups. Then indirectly affect the transcription factors, which were WRKY22 and WRKY25that transcription factor involved in the expression of defense genes in innate immune response of plants, could regulate transcription process. Ultimately, the expression of related defense enzymes (CAT, POD and SOD) were inhibited.

In addition, KEGG Automatic Annotation Server 2.1 (KAAS) (http://www.genome.jp/tools/kaas/) was used to conduct pathway enrichment [46]. Two specific proteins, nucleoside diphosphate kinase (NDPK2) (Q5TKF4_ORYSJ) and catalase (Q9ZRI9_ORYSJ) were found to play important roles in the MAPK signaling cascade pathway. A previous study showed that NDPK2 is a key metabolic enzyme that can maintain the balance of ATP in cells. It was accumulated strongly after infection with *Xanthomonas oryzae* pv. oryzae [47]. The results of the present study showed that NDPK2 was only expressed in the control groups, in which rice was infected with SRBSDV. This result also indicated that NDPK2 is a specifically expressed substance after the rice was infected by the pathogen. NDPK2 indirectly down-regulated mitogen-activated protein kinase (MPK3/6) expression, which can enhance the activities of peroxidase family [48]. Transcription factors of WRKY22 and WRKY25, which are a class of DNA-binding proteins, were indirectly affected by MPK3/6. WRKY transcription factors alleviated oxidative stress tolerance [49,50]. Ultimately, the expression of related defense enzymes (CAT, POD, and SOD) were inhibited, thus NDPK2 can be a marker in SRBSDV-infected rice. However, after COS treatment, the cells produced H_2_O_2_, which induced the specific up-regulated expression of catalase through a kind of phosphate cascade reaction. The catalase then inhibited H_2_O_2_ production and enabled the cells to remove the harmful effects of reactive oxygen species and elicit a stress-tolerant response. This process is shown in Figure 8.

**Figure 9:**
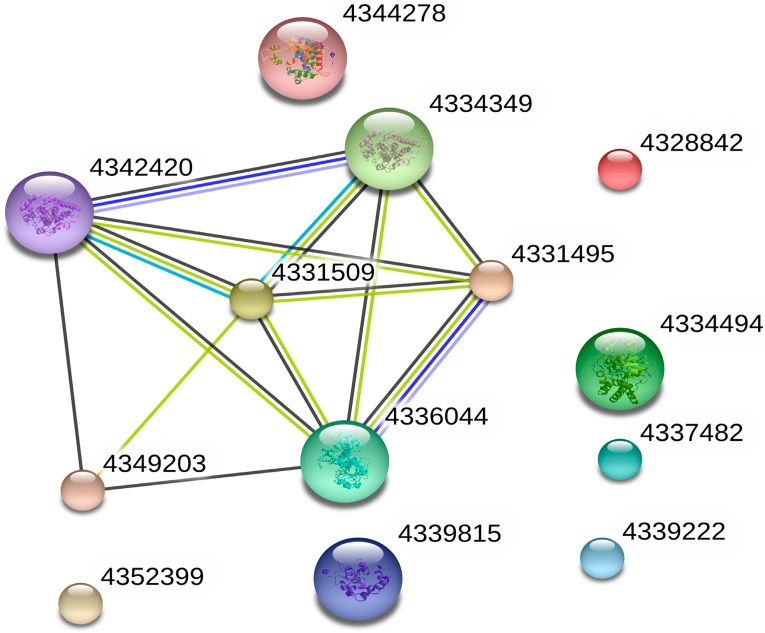
Protein–protein interaction network, catalase interacted with five other whole proteins, namely, glyceraldehyde-3-phosphate dehydrogenase (4336044), glyceraldehyde-3-phosphate dehydrogenase (4331495), hydroxy acid oxidase 1 (4342420), hydroxy acid oxidase 1 (4334349), and glutathione S-transferase (4349203). The interaction among them is related to the regulation of COS.

An essential catalase (4331509) was detected through the protein-protein interaction (PPI) networkin Figure 9. This catalase interacted with five proteins, including glyceraldehyde-3-phosphate dehydrogenase (4336044), glyceraldehyde-3-phosphate dehydrogenase (4331495), hydroxy acid oxidase 1 (4342420), hydroxy acid oxidase 1 (4334349) and glutathione S-transferase (4349203). Previous studies indicated that glyceraldehyde-3-phosphate dehydrogenase enhances tolerance to abiotic stresses through the regulation of H_2_O_2_ levels in rice [51]. The glutathione S-transferase and CAT can increase oxidative stress protection in glutathione *S*-transferase and CAT transgenic rice plants [52]. These results indicated that catalase mainly played essential roles in the regulated resistance of COS. Our enzyme activity tests and gene quantification results indicated that COS promoted defense-related antioxidant enzymes and gene expression both increases. Interestingly, the *PR-1* transcription level increased dramatically. This suggested that COS might also activate rice-associated immune responses through other pathways or ways to resist further replication of the SRBSDV. In Arabidopsis plants, recent studies have shown that COS can induce plants to produce PR-1 protein through the salicylic acid pathway [53]. Our resultsprovide some basis for further study of the COS mechanism.

## 5. Conclusions

In this study, label-free quantitative proteomics, enzyme activity test, and RT-qPCR analysis determined the responses of SRBSDV-infected rice to COS. The bioinformatics analysis results showed that the signal of protecting and controlling the SRBSDV was transmitted through the MAPK signaling cascade pathway. The activities of the defensive enzymes (POD, CAT, and SOD) were activated in COS-treated rice. Simultaneously, the expression levels of the defense genes were increased. The results indicated that NDPK is a signal protein for viral invasion of rice, and catalase is a key regulatory enzyme that can enhance the resistance of rice against SRBSDV. The results of the present study provided a basis for further study of plant immune activation mechanism through COS.

## Figures and Tables

**Table 1:** Primer sequences and the reaction condition used in reverse transcription (RT)-quantitative PCR.

Gene Name	Forward Primer	Reverse Primer
*β-actin*	5′-TCCTCCGTGGAGAAGAGCTA-3′	5′-GCAATGCCAGGGAACATAGT-3′
*Chitinase*	5′-CTACACGTACGACGCCTTCA-3′	5′-GAAGCAGTAACCCCACTGGA-3′
*Catalase*	5′-AAGCCGAGCATGTAAGGAGA-3′	5′-ACACGAATTGTGCGGTGATA-3′
*Ascorbate POD*	5′-CAAGGAGGAGATACCCACCA-3′	5′-TAGGTGGTCAGAACCCTTGG-3′
*PR-1*	5′-CAGTGGTACGACCACGACAG-3′	5′-GGCGAGTAGTTGCAGGTGAT-3′
*Copper/Zine SOD*	5′-TTTTCCAGTCCCCTTCCTCT-3′	5′-AGCCGTGAAGTCCAGGAGTA-3′

POD: Peroxidase; PR-1: pathogenesis related protein 1; SOD: Superoxide Dismutase.

**Table 2:** Chitosan oligosaccharide (COS) at different concentrations to promote rice germination results.

	**Buds Long**							
**Repeated Numbers**	1	2	3	4	5	6	7	**Average Value**
CK	1.6	1.5	1.4	1.3	0.9	0.9	0.9	1.21 ± 0.39
COS(50µg/mL)	2.5	2.4	3.0	2.4	2.0	2.0	2.7	2.43 ± 0.33
COS (100µg/mL)	1.8	1.7	1.5	2.0	1.8	1.5	1.5	1.69 ± 0.18
COS (200µg/mL)	3.5	3.0	4.0	2.5	2.5	2.0	2.5	2.86 ± 0.64
	**Root Long**							
**Repeated Numbers**	1	2	3	4	5	6	7	**Average Value**
CK	1.4	1.7	2.0	2.3	2.0	1.7	2.2	1.90 ± 0.29
COS (50µg/mL)	2.5	2.6	4.0	3.0	3.1	3.6	3.5	3.19 ± 0.51
COS (100µg/mL)	4.0	2.5	2.5	4.0	2.0	3.5	2.5	3.00 ± 0.76
COS (200µg/mL)	1.5	1.8	2.0	1.8	2.0	2.0	2.0	1.87 ± 0.17

CK: control check groups that were treated with water.

**Table 3:** Major different proteins annotated results. After COS treatment, we qualify to defense-related proteins and reduced expression of SRBSDV P9-1, an α-helical protein, self-interacts and forms viroplasms in vivo, P5that is a non-structural protein targeted to chloroplasts, and major capsid protein; results demonstrated that COS can decrease the infection ability of SRBSDV or inhibit the replication of the SRBSDV and improve rice disease resistance. The value of sig represents the different expression. 1 is up-regulation, −1 is down-regulated, 0 means only in the control groups or treatment groups expression or no change in the two groups.

Protein ID	Protein Names	Organism	Length	LogRatio	LogP	Sig
PP2A2_ORYSJ	Serine/threonine-protein phosphatase PP2A-2 catalytic subunit (EC 3.1.3.16)	*Oryza sativa* subsp. *Japónica* (Rice)	307	2.71344	1.809716	1
GLO1_ORYSJ	Peroxisomal (*S*)-2-hydroxy-acid oxidase GLO1 (EC 1.1.3.15) (Glycolate oxidase 1) (GOX 1) (OsGLO1) (Short chain alpha-hydroxy acid oxidase GLO1)	*Oryza sativa* subsp. *japonica* (Rice)	369	1.100617	3.704594	1
GLO5_ORYSJ	Peroxisomal (*S*)-2-hydroxy-acid oxidase GLO5 (EC 1.1.3.15) (Glycolate oxidase 5) (GOX 5) (OsGLO5) (Short chain alpha-hydroxy acid oxidase GLO5)	*Oryza sativa* subsp. *Japónica* (Rice)	369	0.68682	2.115814	1
PER1_ORYSJ	Peroxidase 1 (EC 1.11.1.7)	*Oryza sativa* subsp. *Japónica* (Rice)	326	1.197095	2.103695	1
V5LEK5_9REOV	P9-1	*Southern rice black-streaked dwarf virus*	347	−3.45753	2.791139	−1
Q9ZRI9_ORYSJ	Catalase (EC 1.11.1.6)	*Oryza sativa* subsp. *Japónica* (Rice)	492	0.883992	2.928169	1
Q7XSV2_ORYSJ	Peroxidase (EC 1.11.1.7)	*Oryza sativa* subsp. *Japónica* (Rice)	346	1.18305	1.649601	1
Q5U1F5_ORYSJ	Peroxidase (EC 1.11.1.7)	*Oryza sativa* subsp. *Japónica* (Rice)	344	−1.7243	1.38769	−1
H9BJU7_9REOV	P5	*Southern rice black-streaked dwarf virus*	939	−4.47428	2.610644	−1
Q6ER49_ORYSJ	Peroxidase (EC 1.11.1.7)	*Oryza sativa* subsp. *japonica* (Rice)	321	0.899285	1.336709	1
Q7F1U0_ORYSJ	Peroxidase (EC 1.11.1.7)	*Oryza sativa* subsp. *japonica* (Rice)	317	−0.97083	2.274778	−1
Q9SNK3_ORYSJ	Glyceraldehyde-3-phosphate dehydrogenase (EC 1.2.1.-)	*Oryza sativa* subsp. *Japónica* (Rice)	444	0.75745	2.954426	1
Q7X8A1_ORYSJ	Glyceraldehyde-3-phosphate dehydrogenase (EC 1.2.1.-)	*Oryza sativa* subsp. *Japónica* (Rice)	402	0.94783	2.074914	1
Q945W5_ORYSJ	Glutathione S-transferase GSTU6, putative, expressed (Os10g0530500 protein) (Putative glutathione S-transferase) (Putative glutathione S-transferase OsGSTU13)	*Oryza sativa* subsp. *Japónica* (Rice)	233	1.129885	2.872945	1
Q5TKF4_ORYSJ	Nucleoside diphosphate kinase (EC 2.7.4.6)	*Oryza sativa* subsp. *Japónica* (Rice)	239	-	-	0

P9-1: it is an α-helical protein, it self-interacts and forms viroplasms in vivo. P5: it is a non-structural protein of SRBSDV targeted to chloroplasts.

## Supplementary Materials

The following are available online at www.mdpi.com/1999-4915/9/5/115/s1, Table S1: Identification of total protein results, Table S2: Differentially expressed proteins of rice in Treatment/control, Table S3: Results of differential protein GO analysis.

## References

[1] Zhang H.M., Yang J., Chen J.P., Adams M. (2008). A black-streaked dwarf disease on rice in China is caused by a novel fijivirus. Arch. Virol..

[2] Zhou G., Wen J., Cai D., Li P., Xu D., Zhang S. (2008). Southern rice black-streaked dwarf virus: A new proposed *Fijivirus* species in the family *Reoviridae*. Chin. Sci. Bull..

[3] Xu H.X., Zheng X.S., Yang Y.J., Tian J.C., Lu Y.H., Tan K.H., Heong K.L., Lu Z.X. (2015). Methyl eugenol bioactivities as a new potential botanical insecticide against major insect pests and their natural enemies on rice (*Oriza sativa*). Crop Protect..

[4] Younes I., Rinaudo M. (2015). Chitin and Chitosan Preparation from Marine Sources. Structure, Properties and Applications. Mar. Drugs.

[5] Hadwiger L.A., Beckman J.M. (1980). Chitosan as a component of pea-*Fusarium solani* interactions. Plant Physiol..

[6] Jones J.D., Dangl J.L. (2006). The plant immune system. Nature.

[7] Chisholm S.T., Coaker G., Day B., Staskawicz B.J. (2006). Host-microbe interactions: shaping the evolution of the plant immune response. Cell.

[8] Yin H., Zhao X., Du Y. (2010). Oligochitosan: A plant diseases vaccine—A review. Carbohydr. Polym..

[9] Zhang H., Wang W., Yin H., Zhao X., Du Y. (2012). Oligochitosan induces programmed cell death in tobacco suspension cells. Carbohydr. Polym..

[10] Wang M., Chen Y., Zhang R., Wang W., Zhao X., Du Y., Yin H. (2015). Effects of chitosan oligosaccharides on the yield components and production quality of different wheat cultivars (*Triticum*
*aestivum* L.) in Northwest China. Field Crops Res..

[11] Li S.-J., Zhu T.-H. (2013). Biochemical response and induced resistance against anthracnose (*Colletotrichum*
*camelliae*) of camellia (*Camellia pitardii*) by chitosan oligosaccharide application. For. Pathol..

[12] Yin H., Li Y., Zhang H.Y., Wang W.X., Lu H., Grevsen K., Zhao X., Du Y. (2013). Chitosan Oligosaccharides–Triggered Innate Immunity Contributes to Oilseed Rape Resistance against *Sclerotinia*
*Sclerotiorum*. Int. J. Plant Sci..

[13] Zhang P., Chen K. (2009). Age-dependent variations of volatile emissions and inhibitory activity toward *Botrytis cinerea* and *Fusarium oxysporum* in tomato leaves treated with chitosan oligosaccharide. J. Plant Biol..

[14] Cabrera J.C., Messiaen J., Cambier P., van Cutsem P. (2006). Size, acetylation and concentration of chitooligosaccharide elicitors determine the switch from defence involving PAL activation to cell death and water peroxide production in Arabidopsis cell suspensions. Physiol. Plant..

[15] Falcón A.B., Cabrera J.C., Costales D., Ramírez M.A., Cabrera G., Toledo V., Martínez-Téllez M.A. (2008). The effect of size and acetylation degree of chitosan derivatives on tobacco plant protection against *Phytophthora parasitica*
*nicotianae*. World J. Microbiol. Biotechnol..

[16] Liénart Y., Gautier C., Domard A. (1991). Isolation from Rubus cell-suspension cultures of a lectin specific for glucosamine oligomers. Planta.

[17] Lee S., Choi H., Suh S., Doo I.S., Oh K.Y., Choi E.J., Taylor A.T.S., Low P.S., Lee Y. (1999). Oligogalacturonic acid and chitosan reduce stomatal aperture by inducing the evolution of reactive oxygen species from guard cells of tomato and *Commelinacommunis*. Plant Physiol..

[18] Vasconsuelo A., Marí A., Picotto G., Rodriguez-Talou J., Boland R. (2003). Involvement of the PLC/PKC pathway in chitosan-induced anthraquinone production by *Rubiatinctorum* L. cell cultures. Plant Sci..

[19] Vasconsuelo A., Marí A., Boland R. (2004). Signal transduction events mediating chitosan stimulation of anthraquinone synthesis in *Rubiatinctorum*. Plant Sci..

[20] Vasconsuelo A., Morelli S., Picotto G., Giulietti A.M., Boland R. (2005). Intracellular calcium mobilization: A key step for chitosan-induced anthraquinone production in *Rubiatinctorum* L.. Plant Sci..

[21] Agrawal G.K., Rakwal R., Tamogami S., Yonekura M., Kubo A., Saji H. (2002). Chitosan activates defense/stress response(s) in the leaves of *Oryza sativa* seedlings. Plant Physiol. Biochem..

[22] Boonlertnirun S., Boonraung C., Suvanasara R. (2008). Application of chitosan in rice production. J. Met. Mater. Miner..

[23] El Hadrami A., Adam L.R., El Hadrami I., Daayf F. (2010). Chitosan in plant protection. Mar. Drugs.

[24] Liu J.P., Tang T., Zhao M.P. (2015). Control efficacy on southern rice black-streaked dwarf virus of oligosaccharins·plant activator protein and its effects on growth promotion and yield increase of rice. Agrochemicals.

[25] Peters R.J. (2006). Uncovering the complex metabolic network underlying diterpenoid phytoalexin biosynthesis in rice and other cereal crop plants. Phytochemistry.

[26] Bradford M.M. (1976). A rapid and sensitive method for the quantitation of microgram quantities of protein utilizing the principle of protein-dye binding. Anal. Biochem..

[27] Sengupta D., Kannan M., Reddy A.R. (2011). A root proteomics-based insight reveals dynamic regulation of root proteins under progressive drought stress and recovery in *Vigna radiata* (L.) Wilczek. Planta.

[28] Cox J., Mann M. (2008). MaxQuant enables high peptide identification rates, individualized p.p.b.-range mass accuracies and proteome-wide protein quantification. Nat. Biotechnol..

[29] Cox J., Neuhauser N., Michalski A., Scheltema R.A., Olsen J.V., Mann M. (2011). Andromeda: A peptide search engine integrated into the MaxQuant environment. J. Proteome Res..

[30] Consortium G.O. (2004). The Gene Ontology (GO) database and informatics resource. Nucleic Acids Res..

[31] Ashburner M., Ball C.A., Blake J.A., Botstein D., Butler H., Cherry J.M., Davis A.P., Dolinski K., Dwight S.S., Eppig J.T. (2000). Gene Ontology: Tool for the unification of biology. Nat. Genet..

[32] Götz S., García-Gómez J.M., Terol J., Williams T.D., Nagaraj S.H., Nueda M.J., Robles M., Talón M., Dopazo J., Conesa A. (2008). High-throughput functional annotation and data mining with the Blast2GO suite. Nucleic Acids Res..

[33] Kanehisa M., Goto S., Sato Y., Furumichi M., Tanabe M. (2012). KEGG for integration and interpretation of large-scale molecular data sets. Nucleic Acids Res..

[34] Szklarczyk D., Morris J.H., Cook H., Kuhn M., Wyder S., Simonovic M., Santos A., Doncheva N.T., Roth A., Bork P. (2017). The STRING database in 2017: quality-controlled protein-protein association networks, made broadly accessible. Nucleic Acids Res..

[35] Livak K.J., Schmittgen T.D. (2001). Analysis of relative gene expression data using real-time quantitative PCR and the 2^−ΔΔCT^ method. Methods.

[36] Huang D.W., Sherman B.T., Lempicki R.A. (2009). Bioinformatics enrichment tools: Paths toward the comprehensive functional analysis of large gene lists. Nucleic Acids Res..

[37] Huang D.W., Sherman B.T., Lempicki R.A. (2009). Systematic and integrative analysis of large gene lists using DAVID bioinformatics resources. Nat. Protoc..

[38] Thissen D., Steinberg L., Kuang D. (2002). Quick and easy implementation of the Benjamini-Hochberg procedure for controlling the false positive rate in multiple comparisons. J. Educ. Behav. Stat..

[39] Benjamini Y., Hochberg Y. (1995). Controlling the False Discovery Rate: A Practical and Powerful Approach to Multiple Testing. J. R. Stat. Soc. B.

[40] Shah K., Nahakpam S. (2012). Heat exposure alters the expression of SOD, POD, APX and CAT isozymes and mitigates low cadmium toxicity in seedlings of sensitive and tolerant rice cultivars. Plant Physiol. Biochem..

[41] Li M., Wang G. (2001). Effect of Drought Stress on Activities of Cell Defense Enzymes and Lipid Peroxidation in *Glycyrrhiza*
*uralensis* Seedings. Acta Ecol. Sin..

[42] Zhang C., Liu Y., Liu L., Lou Z., Zhang H., Miao H., Hu X., Pang Y., Qiu B. (2008). Rice black streaked dwarf virus P9–1, an α-helical protein, self-interacts and forms viroplasms in vivo. J. Gen. Virol..

[43] Liu X.Y., Yang J., Xie L., Li J., Song X.J., Chen J.P., Zhang H.M. (2015). P5–2 of rice black-streaked dwarf virus is a non-structural protein targeted to chloroplasts. Arch. Virol..

[44] Li J., Xue J., Zhang H.-M., Yang J., Xie L., Chen J.-P. (2015). Characterization of homologous and heterologous interactions between viroplasm proteins P6 and P9–1 of the fijivirus southern rice black-streaked dwarf virus. Arch. Virol..

[45] Sun L., Xie L., Andika I.B., Tan Z., Chen J. (2013). Non-structural protein P6 encoded by rice black-streaked dwarf virus is recruited to viral inclusion bodies by binding to the viroplasm matrix protein P9–1. J. Gen. Virol..

[46] Moriya Y., Itoh M., Okuda S., Yoshizawa A.C., Kanehisa M. (2007). KAAS: An automatic genome annotation and pathway reconstruction server. Nucleic Acids Res..

[47] Cho S.M., Shin S.H., Kim K.S., Kim Y.C., Eun M.Y., Cho B.H. (2004). Enhanced expression of a gene encoding a nucleoside diphosphate kinase 1 (OsNDPK1) in rice plants upon infection with bacterial pathogens. Mol. Cells.

[48] Yao S., Wang Y., Yang T., Kou F., Lu W., Xiao K. (2016). Expression pattern and function of wheat mitogen-activated protein kinase (MPK) cascade genes under micronutrient-deprived conditions. Acta Physiol. Plant..

[49] Wang X., Zeng J., Li Y., Rong X., Sun J., Sun T., Li M., Wang L., Feng Y., Chai R. (2015). Expression of *TaWRKY44*, a wheat *WRKY* gene, in transgenic tobacco confers multiple abiotic stress tolerances. Front. Plant Sci..

[50] Gong X.Q., Hu J.B., Liu J.H. (2014). Cloning and characterization of *FcWRKY40*, A WRKY transcription factor from *Fortunella*
*crassifolia* linked to oxidative stress tolerance. Plant Cell Tissue Organ Cult..

[51] Zhang X.H., Rao X.L., Shi H.T., Li R.J., Lu Y.T. (2011). Overexpression of a cytosolic glyceraldehyde-3-phosphate dehydrogenase gene *OsGAPC3* confers salt tolerance in rice. Plant Cell Tissue Organ Cult..

[52] Zhao F., Zhang H. (2006). Salt and paraquat stress tolerance results from co-expression of the *Suaeda salsa* glutathione S-transferase and catalase in transgenic rice. Plant Cell Tissue Organ Cult..

[53] Jia X., Meng Q., Zeng H., Wang W., Yin H. (2016). Chitosan oligosaccharide induces resistance to Tobacco mosaic virus in Arabidopsis via the salicylic acid-mediated signalling pathway. Sci. Rep..

